# ASPM stabilizes the NOTCH intracellular domain 1 and promotes oncogenesis by blocking FBXW7 binding in hepatocellular carcinoma cells

**DOI:** 10.1002/1878-0261.13589

**Published:** 2024-01-26

**Authors:** Tze‐Sian Chan, Li‐Hsin Cheng, Chung‐Chi Hsu, Pei‐Ming Yang, Tai‐Yan Liao, Hsiao‐Yen Hsieh, Pei‐Chun Lin, Wei‐Chun HuangFu, Chih‐Pin Chuu, Kelvin K. Tsai

**Affiliations:** ^1^ Laboratory of Advanced Molecular Therapeutics, Graduate Institute of Clinical Medicine, College of Medicine Taipei Medical University Taiwan; ^2^ Division of Gastroenterology, Department of Internal Medicine, Wan Fang Hospital Taipei Medical University Taiwan; ^3^ School of Medicine, College of Medicine Taipei Medical University Taiwan; ^4^ Pancreatic Cancer Group, Taipei Cancer Center Taipei Medical University Taiwan; ^5^ Core Laboratory of Organoids Technology, Office of R&D Taipei Medical University Taiwan; ^6^ School of Medicine, College of Medicine I‐Shou University Kaohsiung City Taiwan; ^7^ Master Program in Graduate Institute of Cancer Biology and Drug Discovery Taipei Medical University Taiwan; ^8^ Institute of Cellular and System Medicine National Health Research Institutes Miaoli Taiwan; ^9^ TMU Research Center of Cancer Translational Medicine Taipei Medical University Taiwan

**Keywords:** ASPM, FBXW7, hepatocellular carcinoma, Notch signaling, NOTCH1, tumorigenesis

## Abstract

Notch signaling is aberrantly activated in approximately 30% of hepatocellular carcinoma (HCC), significantly contributing to tumorigenesis and disease progression. Expression of the major Notch receptor, *NOTCH1*, is upregulated in HCC cells and correlates with advanced disease stages, although the molecular mechanisms underlying its overexpression remain unclear. Here, we report that expression of the intracellular domain of NOTCH1 (NICD1) is upregulated in HCC cells due to antagonism between the E3‐ubiquitin ligase F‐box/WD repeat‐containing protein 7 (FBXW7) and the large scaffold protein abnormal spindle‐like microcephaly‐associated protein (ASPM) isoform 1 (ASPM‐i1). Mechanistically, FBXW7‐mediated polyubiquitination and the subsequent proteasomal degradation of NICD1 are hampered by the interaction of NICD1 with ASPM‐i1, thereby stabilizing NICD1 and rendering HCC cells responsive to stimulation by Notch ligands. Consistently, downregulating ASPM‐i1 expression reduced the protein abundance of NICD1 but not its FBXW7‐binding‐deficient mutant. Reinforcing the oncogenic function of this regulatory module, the forced expression of NICD1 significantly restored the tumorigenic potential of ASPM‐i1‐deficient HCC cells. Echoing these findings, NICD1 was found to be strongly co‐expressed with ASPM‐i1 in cancer cells in human HCC tissues (*P* < 0.001). In conclusion, our study identifies a novel Notch signaling regulatory mechanism mediated by protein–protein interaction between NICD1, FBXW7, and ASPM‐i1 in HCC cells, representing a targetable vulnerability in human HCC.

## Graphical abstract

The upregulated expression of the oncogenic isoform 1 of ASPM (ASPM‐i1) in hepatocellular carcinoma (HCC) correlates with pathologic grade and poor patient survival. ASPM‐i1 hampers the FBXW7‐mediated polyubiquitination and degradation of NOTCH1 intracellular domain (NICD1), thereby rendering HCC cells responsive to stimulation by Notch ligands. The novel Notch‐activating mechanism represents a targetable vulnerability in human HCC.

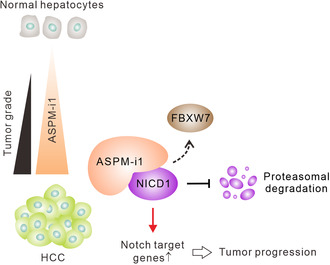

**Abbreviations**
ASPM: abnormal spindle‐like microcephaly‐associated protein
ASPM‐i1: abnormal spindle‐like microcephaly‐associated protein isoform 1
*ASPM*‐v1: *ASPM* transcript variant 1
BLI: bioluminescence images
CDS: coding sequence
CHX: cycloheximide
CoA: coactivator
CSL: CBF1, Suppressor of Hairless, Lag‐1
DLL: Delta‐like ligand
DVL: dishevelled
FBS: fetal bovine serum
FBXW7: F‐box/WD repeat‐containing protein 7
FF‐Luc: firefly luciferase
GFP: green fluorescence protein
GSEA: gene set enrichment analyses
HCC: hepatocellular carcinoma
IB: immunoblotting
IF: immunofluorescence
IHC: immunohistochemistry
IP: immunoprecipitation
JAG: Jagged
LDA: limiting‐dilution assay
MAML: Mastermind‐like
NICD1: NOTCH1 intracellular domain
PD‐L1: programmed cell death ligand 1
PI: propidium iodide
qRT‐PCR: quantitative reverse transcription PCR
shRNA: small hairpin RNA
STR: short tandem repeat
Ub: ubiquitin
VEGF: vascular endothelial growth factor

## 1 Introduction

Hepatocellular carcinoma (HCC), a leading cause of cancer‐related mortality worldwide [1], develops stepwise from chronic liver inflammation, cirrhosis to malignant tumors. The prognosis of patients with unresectable HCC is poor as available treatments are limited. Although multi‐kinase inhibitors, including sorafenib and lenvatinib [2], were approved to treat unresectable HCC, they only provide a small survival benefit to patients. New therapeutics such as immune checkpoint inhibitors and anti‐vascular endothelial growth factor (VEGF) antibodies failed to improve patient survival as single agents [3]. It was not until recently that the combination therapy of anti‐programmed cell death ligand 1 (PD‐L1) and anti‐VEGF antibodies was approved as the first‐line therapy for unresectable or metastatic HCC [4]. Still, the survival benefit of this new treatment is only modest, with a survival time of 5.8 months longer than that associated with the use of sorafenib alone. Thus, the satisfactory treatment of advanced HCC remains a highly unmet clinical need, calling for an in‐depth understanding of the molecular mechanisms driving tumorigenesis and malignant progression [5].

Notch signaling is aberrantly activated in various human solid tumors, and convincing evidence has associated dysregulated Notch signaling with malignant progression [6, 7]. Notch signaling contributes to tumorigenesis by regulating diverse oncogenic mechanisms, including cell cycle progression, apoptosis, angiogenesis, epithelial–mesenchymal transition, and cancer stemness [7, 8, 9]. In HCC, Notch signaling plays an important role in tumor development and progression by regulating tumor microenvironment, angiogenesis, invasion, and metastasis [10, 11]. For instance, based on a gene signature reflecting the Notch pathway activity, it has been reported that approximately 30% of human HCC are Notch signaling active. Consistently, the functional inhibition of Notch signaling could reduce the viability and the proliferative potential of HCC cells [12]. The transcript level of *NOTCH1*, which encodes the major Notch pathway receptor, as well as the protein abundance level of its cleaved and transcriptionally active form, NOTCH1 intracellular domain (NICD1), markedly increase in HCC tissues, and their expression levels correlated with advanced disease stages, vascular invasion, and poor overall survival [11, 13, 14]. Despite these reports, there is little evidence of genetic alterations and somatic mutations in the Notch pathway in human HCC [7, 15]. Thus, the molecular mechanisms underlying Notch activation and NOTCH1 overexpression in HCC remain unclear and await further investigation.

Assembly Factor for Spindle Microtubules (ASPM) is a key regulator of neurogenesis and brain size and is widely expressed in normal or malignant human tissues [16, 17]. There is now a growing body of evidence suggesting the oncogenic role of ASPM, specifically its isoform 1 (ASPM‐i1), in diverse malignant tumors by augmenting cellular Wnt responsiveness [18, 19, 20, 21, 22, 23, 24]. In HCC, ASPM is overexpressed in approximately 66% of tumors [25], and its expression level has been associated with tumor grades and poor patient prognosis [24]. Mechanistically, ASPM regulates the stemness of HCC cells by stabilizing the upstream Wnt regulator disheveled (DVL) proteins, thereby augmenting Wnt‐β‐catenin signaling [20].

Here, we reported a novel Notch regulatory mechanism in HCC cells mediated by the protein–protein interaction between NICD1, its E3‐ubiquitin ligase FBXW7 and ASPM‐i1. This module stabilizes NICD1 to facilitate the expression of Notch target genes and contributes to HCC tumorigenesis. The novel mechanistic insight into Notch pathway activation in human HCC provided by our study may have important prognostic and therapeutic implications.

## 2 Materials and methods

### 2.1 Cell culture

Primary HCC KVGH‐80T (non‐hepatitis B or hepatitis C‐related) and KVGH‐90T (hepatitis C‐related) cells (gift of Dr. Hung‐Wei Pan, I‐Shou University) were isolated and cultivated as described previously [20]. HuH‐1 (CVCL_2956), SNU‐449 (CVCL_0454), SNU‐475 (CVCL_0497), and HuH‐7 (CVCL_0336) cells (both from the Japanese Collection of Research Bioresources) were propagated on tissue culture plastics in RPMI‐1640 or DMEM (Invitrogen, Waltham, MA, USA) supplemented with 10% fetal bovine serum (FBS). Normal human hepatocytes were isolated from the human liver and cultured in Hepatocyte Medium according to the supplier's recommendation (ScienCell Research Laboratories, Carlsbad, CA, USA). All of the cells had the short tandem repeat (STR) authentication, and all experiments were performed with mycoplasma‐free cells.

### 2.2 Immunoblotting (IB) and co‐immunoprecipitation (IP) analysis

IB and co‐IP analyses were performed according to standard protocols described previously [18]. The rabbit polyclonal “pan‐isoform” anti‐ASPM antibody and antibodies specifically detecting ASPM‐i1 (NCBI RefSeq: NP_060606.3) or ASPM‐i2 (NCBI RefSeq: NP_001193775.1) were developed by our group as described previously [18]. A goat anti‐rabbit IgG (Jackson ImmunoResearch, West Grove, PA, USA) was used in conjunction with the polyclonal antibodies for the immune detection of ASPM isoforms as described above. Other primary antibodies used are listed in Table S1.

### 2.3 Bioinformatics analysis

The RNA sequencing transcript level of *ASPM* transcript variant 1 (*ASPM*‐v1; NCBI RefSeq: NM_018136.4; ENST00000367409.9; encoding ASPM‐i1) in normal liver and HCC tissues, along with the patient survival data in The Cancer Genome Atlas–liver hepatocellular carcinoma (TCGA‐LIHC) data set were interrogated from the GEPIA2 database (http://gepia2.cancer‐pku.cn/#index).

### 2.4 Quantitative reverse transcription PCR (qRT‐PCR) analysis

qRT‐PCR analysis was performed on the amplified RNA using the LightCycler FastStart DNA MASTERPLUS SYBR Green I Kit and the LightCycler System (Roche Diagnostics GmbH, Mannheim, Germany) according to the manufacturer's instructions. Oligonucleotide primers were designed using Primer Bank (http://pga.mgh.harvard.edu/primerbank/index.html).

### 2.5 Immunofluorescence and immunohistochemistry analyses

Immunofluorescence (IF) staining of cells grown on culture plastics was performed using standard protocols and imaged using the VS120 Virtual Slide Microscope (Olympus, Tokyo, Japan). The tissue microarrays of six normal liver tissues and 50 HCC tissues with associated pathological grade data were acquired from TissueArray.Com (#LV2081; Derwood, MD, USA). Immunohistochemistry (IHC) analysis was carried out as described previously [18]. The staining was detected using the DAKO EnVision kit (Agilent Technologies, Santa Clara, CA, USA). The staining was independently evaluated by an expert hepatologist (T.S. Chan) and verified by an expert pathologist (W.Y. Chen, Wan Fang Hospital, Taipei, Taiwan) in a randomized manner. The staining intensities were quantified at the single‐cell level with at least 200 tumor cells counted per tumor (2–3 tissue sections counted per tumor; at least 100 tumor cells counted per section).

### 2.6 Gene expression manipulations

The specific knockdown of *ASPM*‐v1 expression was achieved by lentivirus‐mediated transduction of small hairpin RNA (shRNA) using RNA target sequences (shRNA #4 and #3) specific for exon 18 of the *ASPM* gene (unique to *ASPM*‐v1) as described previously [18]. A non‐targeting oligonucleotide sequence (SHC002V; Sigma‐Aldrich, St. Louis, MO, USA) was used as a control. The details of other gene expression manipulations are described in Supplementary Information. Lentivirus was produced in Lenti‐X 293T™ cells (Clontech/Takara Bio, Mountain View, CA, USA) using the packaging vectors pMD2.G (Addgene plasmid #12259; http://n2t.net/addgene:12259; RRID: Addgene_12259, Watertown, MA, USA) and psPAX2 (Addgene plasmid #12260; http://n2t.net/addgene:12260; RRID: Addgene_12260) to boost viral titer. The coding sequence (CDS) for NICD1 and GFP is PCR amplified from the lentiviral expression vector of human NICD1, EF.hICN1.CMV.GFP (Addgene plasmid #17623; http://n2t.net/addgene:17623; RRID: Addgene_17623) and overlap extension PCR was used to construct a NICD1–GFP fusion gene. The fusion gene product was ligated into the vector to generate EF.hICN1.GFP as the final expression construct. The expression vector for the GFP‐epitope‐tagged NICD1 (T2511A) mutant was constructed using the following primers:

Primer 1: 5′‐CTGTTTCTGGCCGCCCGGGAGG‐3′;
Primer 2: 5′‐AGGGGACGGGGCGAGGAAGGGGTGCTCAGGCAC‐3′;
Primer 3: 5′‐CCTTCCTCGCCCCGTCCCCTGAGTCCCCTGAC‐3′;
Primer 4: 5′‐ACACTGGCGGCCGTTACTAGTGGATCTGAC‐3′.

The PCR product was substituted for the full‐length human NICD1 on EF.hICN1.GFP using the In‐Fusion HD Cloning Kit (Takara Bio, San Jose, CA, USA). The validated small hairpin RNA (shRNA) oligonucleotides (TRCN0000350253 and TRCN0000350330) in the lentivector pLKO.1‐puro used for the sustained knockdown of *NOTCH1* expression was obtained from Sigma‐Aldrich (St. Louis). The lentiviral expression construct of human *DVL1* has been described previously [20]. The coding sequence of human *DVL2* was PCR amplified from a *DVL2* expression vector (Origene #RC202233; Rockville, MD, USA) and subcloned into the pLenti‐His lentivirus vector (Applied Biological Materials, ABM, Richmond, BC, Canada). Lentivirus was produced in Lenti‐X 293T™ cells (Clontech/Takara Bio) using the packaging vectors pMD2.G (Addgene plasmid #12259; http://n2t.net/addgene:12259; RRID: Addgene_12259) and psPAX2 (Addgene plasmid #12260; http://n2t.net/addgene:12260; RRID: Addgene_12260) to boost viral titer.

### 2.7 Tumorsphere formation assays

The tumorsphere formation assay was performed as previously described [26]. For limiting‐dilution assay (LDA), cells were plated in limiting dilution in 24‐well plates in the respective culture media. The presence of spheres was evaluated after 10 days.

### 2.8 Analysis of cell apoptosis

The apoptotic propensity of cells was analyzed by immunostaining the cells with the apoptotic marker cleaved caspase 3 or the Annexin V and the propidium iodide (PI) staining pattern by fluorescence‐activated cell sorting (Annexin V Conjugates for Apoptosis Detection, #A13201 Invitrogen), wherein Annexin V^+^PI^−^ cells and Annexin V^+^PI^+^ cells are considered as early and late apoptotic cells, respectively.

### 2.9 Orthotopic mouse model of HCC progression

HuH‐1 cells were lentivirally transduced with a green fluorescence protein (GFP) and firefly luciferase (FF‐Luc) fusion vector (UBC‐EGFP‐T2A‐Luc; System Biosciences, Palo Alto, CA, USA), and the GFP‐positive cells were sorted. Cells (1 × 10^6^ cells) were inoculated into the left lobe of the liver of 8‐week‐old female nonobese diabetic/severe combined immunodeficiency (NOD.CB17‐*Prkdc* ^*scid*^/NcrCrl, NOD/SCID) mice (BioLASCO Taiwan, Taipei City, Taiwan). The tumor mass and distribution were assessed by bioluminescence (BLI; the IVIS Imaging System, Caliper Life Sciences, Waltham, MA, USA). All mice were housed five per cage in an air‐conditioned vivarium with free access to food and water. Throughout the study, a 12‐h light/dark cycle was maintained with lights on at 8 AM. Protocols and study for animal care and experimentation were approved by the Institutional Animal Care and Use Committee of Taipei Medical University (Taipei City, Taiwan), which adhered to the NIH Guide for the Care and Use of Laboratory Animals. Animal experiments were conducted under the IACUC number (LAC‐2022‐0365).

### 2.10 Luciferase reporter assay

The lentiviral reporter pMuLE_EXPR_CMV‐eGFP_TOP‐NLuc1.1_12GLI‐FLuc_CBF‐GLuc was acquired from Addgene (Addgene plasmid #113862, http://n2t.net/addgene:113862; RRID: Addgene_113862) [27]. HCC cells were lentivirally transduced with the reporter and then stimulated with recombinant human JAG1‐Fc (5 μg·mL^−1^ for 24 h; Sigma‐Aldrich, Steinheim, Germany) or vehicle [28]. The reporter activity was measured using the ONE‐Glo^®^ Luciferase Assay System (Promega, Madison, WI, USA).

### 2.11 Statistical analysis

The spss 10.0 software (SPSS, Chicago, IL, USA) was used for the statistical analysis of our data. A two‐tailed Student's *t* test was used for simple significance testing. Survival curves were generated using the Kaplan–Meier method. The curves were plotted and compared using the log‐rank test using the graphpad prism version 9.0.0 (GraphPad Software, San Diego, CA, USA). The data from the limiting dilution assay was analyzed and plotted using the ELDA software (http://bioinf.wehi.edu.au/software/elda/index.html). The likelihood ratio test and Chi‐square test were used to assess the significance.

## 3 Results

### 3.1 ASPM‐i1 is overexpressed in human HCC and contributes to the tumorigenic potential of HCC cells

**Fig. 1:**
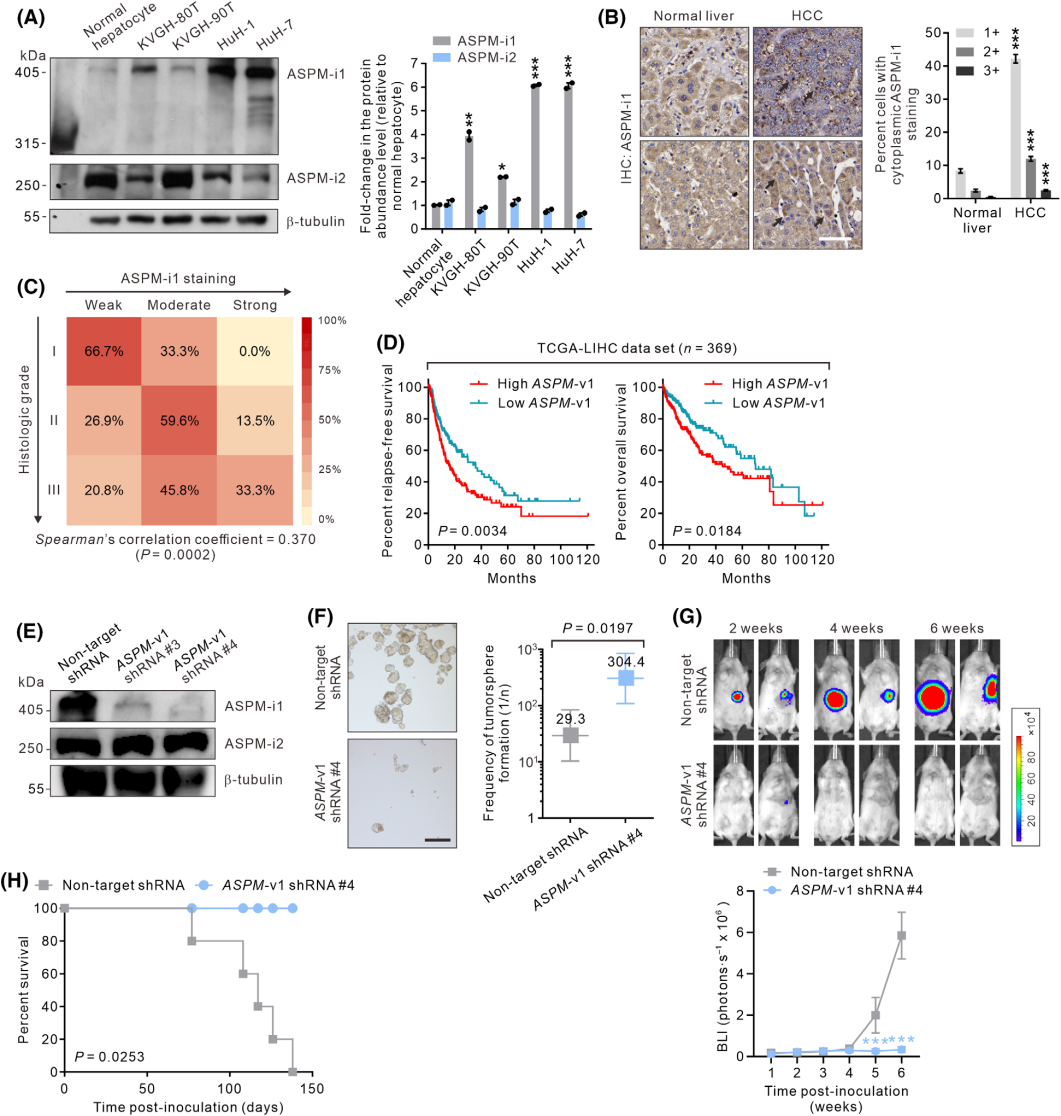
ASPM isoform 1 (ASPM‐i1) is overexpressed in HCC cells and contributes to the Notch signaling pathway responsiveness. (A) Immunoblotting (IB) analysis showing the protein abundance levels of ASPM‐i1 (~ 409 kDa) and ASPM isoform 2 (ASPM‐i2; ~ 250 kDa) in normal hepatocytes, primary HCC cells (KVGH‐80T, KVGH‐90T), and HCC cell lines (HuH‐1, HuH‐7; *n* = 2 independent experiments). β‐tubulin was included as a loading control. The fold‐change in the protein abundance levels of ASPM‐i1 and ASPM‐i2 was quantified by densitometric analysis of the bands (right). (B) Representative immunohistochemical (IHC) staining of ASPM‐i1 in normal liver and HCC tissues (200× magnification). Scale bar denotes 50 μm. Bar graph showing the staining intensities (1+ to 3+) of ASPM‐i1 in the cytoplasm of hepatocytes in normal liver (*n* = 6) and HCC cells in tumor tissues (*n* = 50; right). (C) Heatmap illustrating the correlation of the staining intensity of ASPM‐i1 and the histologic grade of HCC (*n* = 50). (D) Kaplan–Meier survival curve comparing relapse‐free (left) or overall survival (right) of patients with HCC in the TCGA‐LICH data set (*n* = 369). The patients were stratified based on their tumors having a high or low transcript level of *ASPM* variant 1 (*ASPM*‐v1), which encodes ASPM‐i1 (log‐rank test *P* = 0.0034). (E) Immunoblotting (IB) analysis showing the downregulation of ASPM‐i1 expression in primary KVGH‐80T HCC cells lentivirally transduced with *ASPM*‐v1‐targeted shRNA constructs (construct #3 or #4) or a non‐target shRNA. β‐tubulin was included as a loading control (*n* = 2 independent experiments). (F) Representative phase‐contrast images of the tumorspheres formed by KVGH‐80T cells lentivirally transduced with non‐target shRNA or *ASPM*‐v1 shRNA (construct #4). Scale bar denotes 100 μm (left). Limiting dilution assay demonstrating the tumorsphere‐forming efficacy of *ASPM*‐v1 knockdown cells or control (non‐target shRNA) cells (right). Bars represent maximum likelihood estimates with 95% confidence interval (*n* = 6). *P* = 0.0197 by the likelihood ratio test and Chi‐square test. (G) HuH‐1 HCC cells transduced with non‐target shRNA or *ASPM*‐v1 shRNA #4 as in (E) were lentivirally transduced with a firefly luciferase expression vector and then inoculated orthotopically into the left lobe of the liver of NOD/SCID mice. Shown are representative bioluminescence images (BLI) of tumors at the indicated time after cell inoculation. Tumor bulk quantified as BLI normalized photon counts as a function of time (*n* = 5 mice per group; bottom). (H) Percent survival as a function of time in mice described in (G). The *P* value is calculated using the log‐rank test. Data are shown as mean ± SEM. *P < 0.05, **P < 0.01, ***P < 0.001 compared with normal hepatocytes (A), normal liver (B), non‐target shRNA (G), two‐tailed unpaired *t* test.

Previously, we have demonstrated that ASPM‐i1 and ASPM‐i2 are the predominantly expressed ASPM isoforms in pancreatic and gastric cancer cells [18, 19]. We conducted immunoblotting (IB) analysis to profile the expression of ASPM isoforms in human hepatocytes and HCC cells. The data revealed that normal hepatocytes express a very low level of ASPM‐i1, whereas its expression is significantly upregulated in primary HCC cells (KVGH‐80T and KVGH‐90T cells) and established HCC lines (HuH‐1 and HuH‐7 cells) (Fig. 1A). In contrast, the expression of ASPM‐i2, which is likely involved in the housekeeping function of cells [18], was reduced in KVGH‐80T, HuH‐1, and HuH‐7 HCC cells. Given that ASPM‐i1 is the predominantly upregulated isoform in HCC cells, we profiled its expression in human HCC tissues using IHC analysis. The data revealed that hepatocytes in normal liver tissues only exhibit a weak (1+) ASPM‐i1 staining in their cytoplasm (Fig. 1B), while a considerable population of HCC cells displays a moderate (2+; mean 11.9%) or a strong (3+; mean 2.5%) staining intensity of ASPM‐i1. The staining intensity of ASPM‐i1 positively correlates with tumor grade (Spearman's rank correlation coefficient = 0.370; *P* = 0.0002; Fig. 1C). The interrogation into the RNA sequencing data in the TCGA‐LICH data set revealed that the tumors expressing a high transcript level of *ASPM* transcript variant 1 (*ASPM*‐v1; encoding ASPM‐i1) are associated with significantly shorter relapse‐free or overall survival than those with a low expression level (log‐rank *P* = 0.0034; Fig. 1D). To further investigate the functional role of the overexpressed ASPM‐i1 in HCC cells, we stably knocked down the expression of *ASPM*‐v1 in HCC cells using lentivirus‐mediated transduction of shRNA that targets the exon‐18‐encoded mRNA segment specific for *ASPM*‐v1 (Fig. 1E). The specific knockdown of *ASPM*‐v1 expression disabled HCC cells to form tumorspheres under anchorage‐independent conditions, indicating reduced tumorigenic potential (Fig. 1F). Consistently, *ASPM*‐v1 deficiency crippled the ability of HCC cells to form orthotopic liver tumors in immunodeficient NOD/SCID mice (Fig. 1G,H).

### 3.2 ASPM‐i1 contributes to the Notch signaling pathway activity in HCC cells

**Fig. 2:**
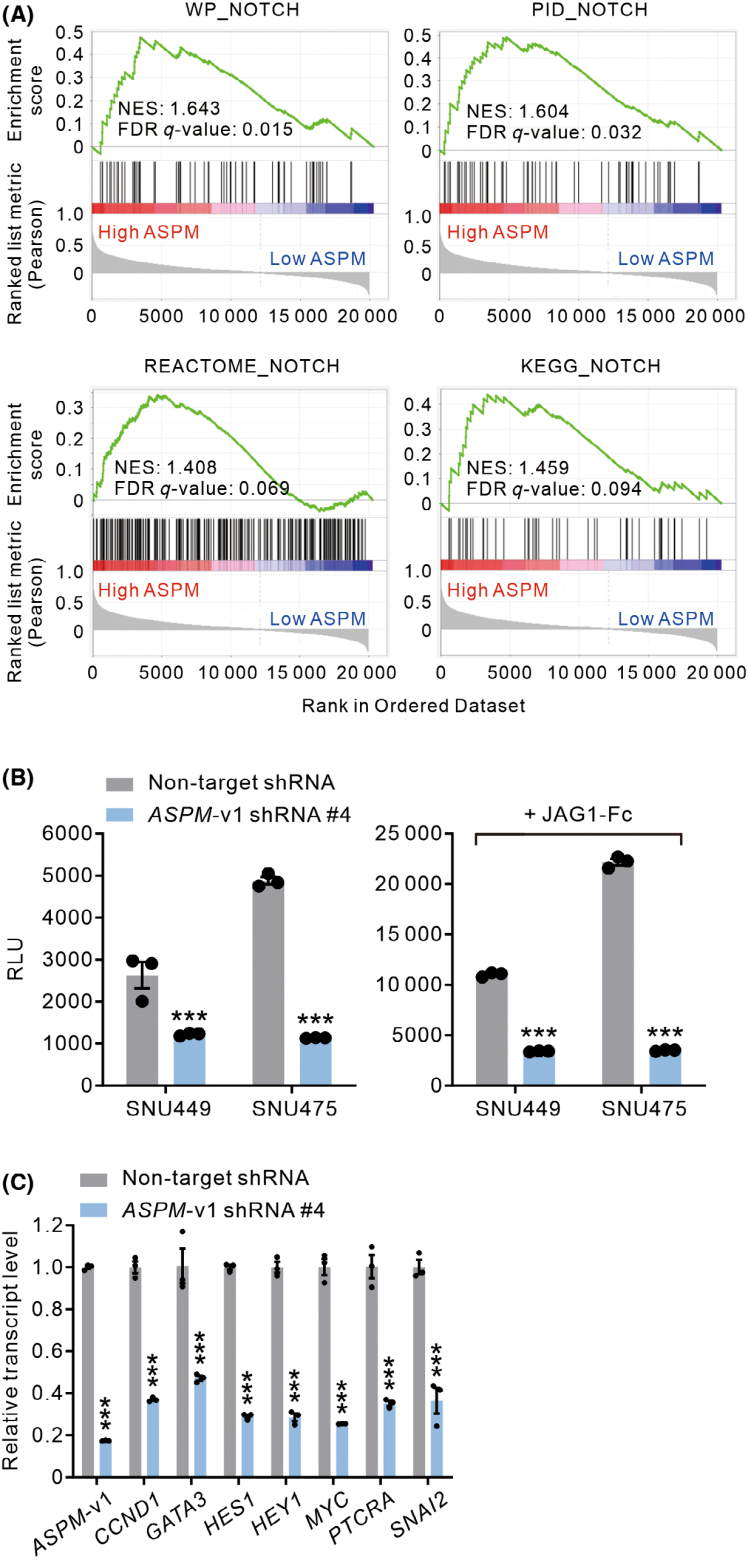
ASPM‐i1 contributes to the Notch signaling pathway activity in HCC cells. (A) Enrichment plots of Gene Set Enrichment Analysis (GSEA) showing that multiple Notch signaling pathway gene sets were significantly enriched in HCC patients with high ASPM expression. NES, normalized enrichment score; FDR, false discovery rate. (B) Notch‐specific luciferase activity (relative light unit; RLU) in SNU‐449 and SNU‐475 cells transduced with non‐target shRNA or *ASPM*‐v1 shRNA (construct #4) without or with stimulation with JAG1‐Fc (5 μg·mL^−1^ for 24 h), (*n* = 3 independent experiments). (C) The relative transcript level of representative Notch target genes in SNU‐449 cells transduced with a non‐target shRNA or *ASPM*‐v1 shRNA (construct #4) following stimulation with JAG1‐Fc as in (B) (*n* = 3 independent experiments). Data are shown as mean ± SEM. ***P < 0.001 compared with non‐target shRNA in (B) and (C), two‐tailed unpaired *t* test.

The development‐associated Notch signaling pathway is activated in a considerable subset of human HCC and contributes to tumor development and progression [10, 11, 12]. The reported roles of ASPM in neurogenesis and development‐associated pathways, such as the Wnt and Hedgehog signaling pathways [29], prompted speculation about its role in the Notch pathway in HCC cells. Consistently, gene set enrichment analyses (GSEA) of the TCGA‐LIHC data set revealed that the Notch pathway gene sets are significantly enriched in patients with HCC with high *ASPM* expression (Fig. 2A). We, thus, asked if ASPM‐i1 contributes to the Notch signaling activity by stably downregulating the expression of *ASPM*‐v1 in the Notch pathway active HCC lines SNU‐449 and SNU‐475 cells using lentivirus‐mediated transduction of shRNA [12]. The knockdown of *ASPM*‐v1 expression significantly lowered the luciferase reporter activity of Notch signaling in these cells (Fig. 2B, left). Moreover, *ASPM*‐v1‐deficient HCC cells exhibited a substantially blunted responsiveness to the Notch ligand JAG1‐Fc (Fig. 2B, right) [28]. Echoing the reduction in the Notch reporter activity, the knockdown of *ASPM*‐v1 expression significantly reduced the transcript levels of representative Notch pathway target genes, including *CCND1*, *GATA3*, *HES1*, *HEY1*, *MYC*, *PTCRA*, and *SNAI2* [7, 9], in JAG1‐Fc‐stimulated SNU‐449 cells (Fig. 2C).

### 3.3 ASPM‐i1 regulates the stability of NICD1 by preventing its interaction with FBXW7

Transcript analysis revealed that the knockdown of *ASPM*‐v1 expression does not markedly affect the transcript levels of the Notch pathway ligands and receptors in HCC cells (Fig. S1A) [30], implying that ASPM‐i1 may not regulate Notch signaling pathway at the transcriptional level. Of note, NOTCH 1 and NOTCH2 are known to be ubiquitously expressed in many tissues, while NOTCH3 and NOTCH4 are mainly expressed in vascular smooth muscle cells and pericytes or endothelial cells [31, 32, 33]. Consistently, the interrogation into the IHC profile of human HCC tissues revealed that NOTCH1 was the predominantly expressed Notch receptor (Fig. S1B).

**Fig. 3:**
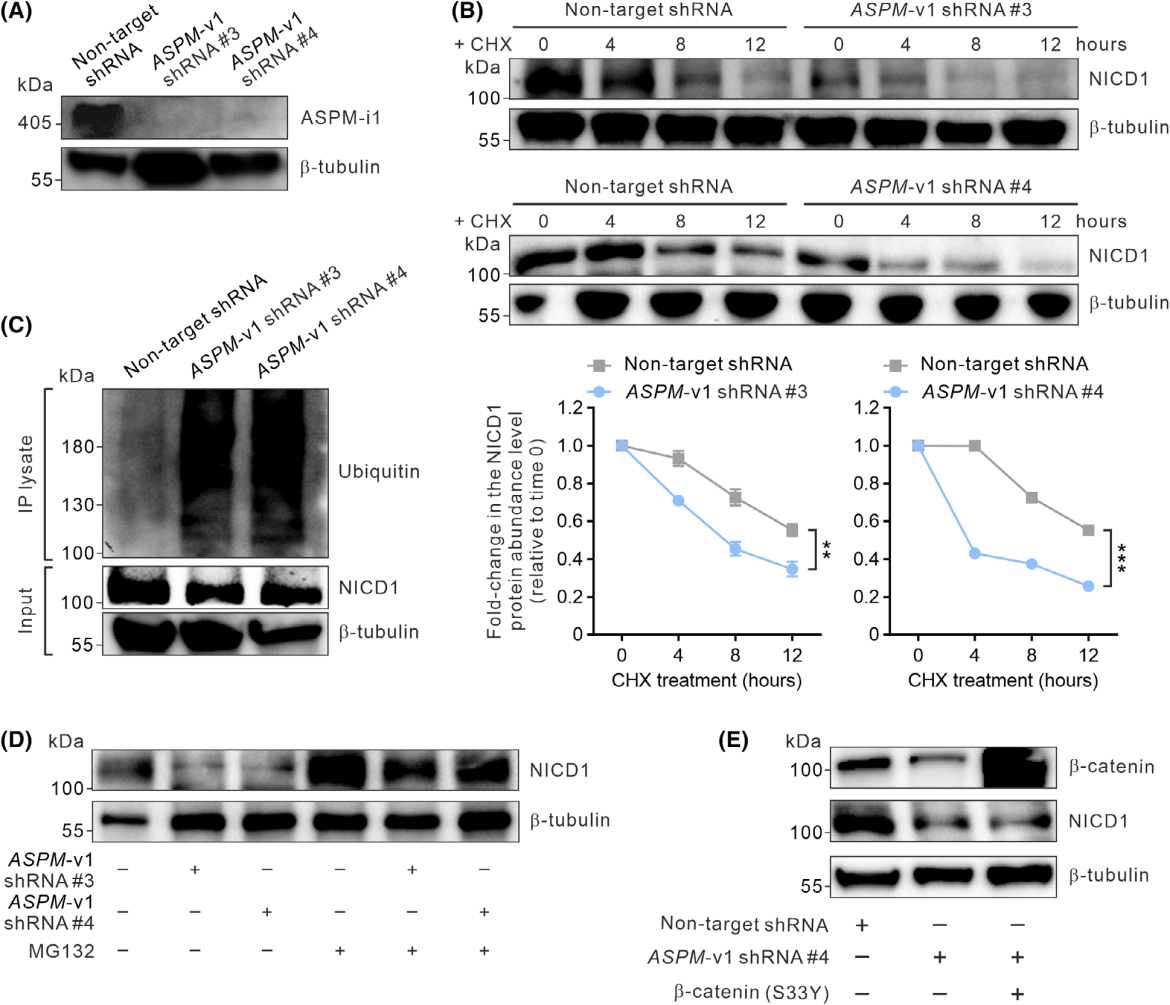
ASPM‐i1 stabilizes NOTCH intracellular domain 1 (NICD1) by preventing its proteasomal degradation in HCC cells. (A) IB analysis showing the effect of shRNA‐mediated knockdown of *ASPM*‐v1 expression in SNU‐449 cells. Two lentivirus shRNA constructs (constructs #3 and #4) were used. β‐tubulin was included as a loading control (*n* = 2 independent experiments). (B) The protein abundance levels of NICD1 in SNU‐449 cells transduced with *ASPM*‐v1 shRNA (construct #3 or #4) or a non‐target shRNA and treated with cycloheximide (CHX) for the indicated length of time (top). Line graphs of the fold‐change in the NICD1 protein abundance levels (bottom). β‐tubulin was included as a loading control (*n* = 2 independent experiments). Data are shown as mean ± SEM. **P < 0.01, ***P < 0.001, ordinary two‐way ANOVA. (C) NICD1 was immunoprecipitated (IP) from SNU‐449 cells without or with the shRNA‐mediated knockdown of *ASPM*‐v1 expression and treated with JAG1‐Fc (5 μg·mL^−1^ for 24 h) and the proteasome inhibitor MG132 (10 μm for 12 h). Poly‐ubiquitinated NICD1 and the total immunoprecipitated protein were detected by immunoblotting (IB). (D) IB analysis showing the effect of knockdown of *ASPM*‐v1 expression on the protein abundance level of NICD1 in SNU‐449 cells following stimulation with JAG1‐Fc without or with MG132 treatment as in (C) (*n* = 2 independent experiments). (E) IB analysis demonstrating that the stable overexpression of active β‐catenin (S33Y) did not affect the protein abundance level of NICD1 in *ASPM*‐v1‐deficient SNU‐449 cells (*n* = 2 independent experiments).

ASPM positively regulates the protein stability of its binding partners, such as cyclin E, DVL, GLI1, and BRCA1, in neuro‐progenitor cells and cancer cells [18, 19, 21, 29, 34, 35]. Since the expression of NICD is under strict regulation by specific E3‐ubiquitin ligases [36, 37], we posited that ASPM‐i1 might regulate the protein stability of NICD1 in HCC cells. Indeed, the knockdown of *ASPM*‐v1 expression diminished the protein abundance level of NICD1 in HCC cells, while the transcript level of *NOTCH1* was unaffected (Fig. S1A,C). To substantiate this finding, we stably knocked down the expression of *ASPM*‐v1 in Notch‐active SNU‐449 cells using two independent shRNA constructs (Fig. 3A). We then treated the cells with the protein translation inhibitor cycloheximide for different lengths of time, by which we uncovered that the half‐life of NICD1 was markedly shortened in *ASPM*‐v1‐deficient cells (Fig. 3B). The reduction in the stability of NICD1 was even more prominent in JAG1‐Fc‐treated cells (Fig. S2). We considered the possibility that ASPM‐i1 might regulate the protein stability of NICD1 by inhibiting its proteasome‐dependent degradation. Indeed, the knockdown of *ASPM*‐v1 expression markedly enhanced the poly‐ubiquitination of NICD1 in SNU‐449 cells (Fig. 3C). As such, treatment of the cells with the proteasome inhibitor MG132 could restore the protein abundance level of NICD1 in *ASPM*‐v1‐deficient cells (Fig. 3D). Of note, although β‐catenin has been shown to regulate the expression of NOTCH1 [38], overexpression of a constitutively active form of β‐catenin did not affect the protein abundance level of NICD1 in *ASPM*‐v1‐deficient HCC cells (Fig. 3E), indicating that ASPM‐i1 regulates NICD1 in a Wnt‐pathway‐independent manner.

**Fig. 4:**
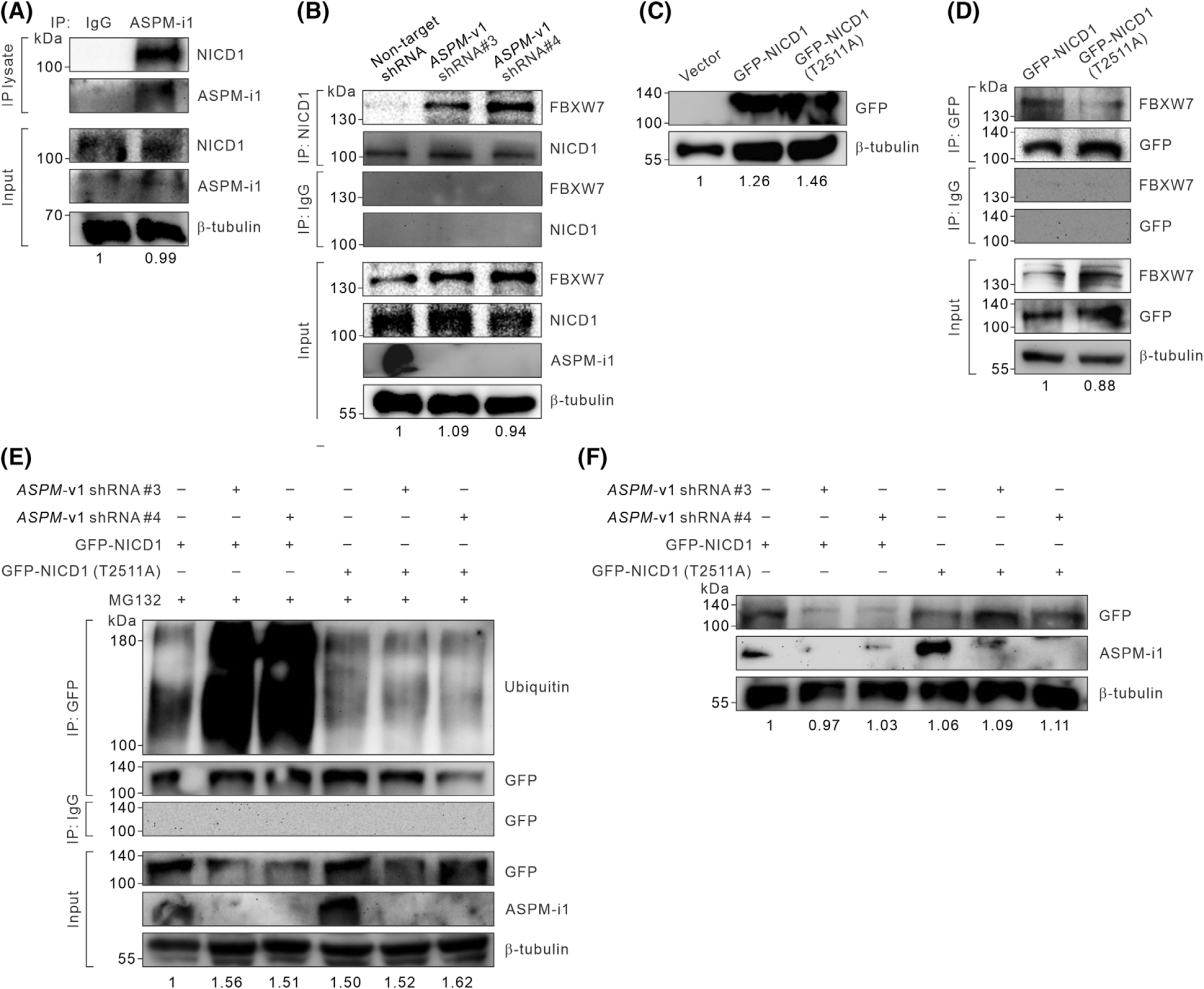
ASPM‐i1 prevents FBXW7‐mediated polyubiquitination of NOTCH1 intracellular domain (NICD1) in HCC cells. (A) Co‐immunoprecipitation (IP) analysis showing that ASPM‐i1 interacts with NICD1 in SNU‐449 cells. (B) Co‐IP analysis demonstrating that the shRNA‐mediated knockdown of *ASPM*‐v1 expression induces the recruitment of FBXW7 to NICD1 in SNU‐449 cells following stimulation with JAG1‐Fc (5 μg·mL^−1^ for 24 h) and treated with MG132 (10 μm for 12 h). (C) SNU‐449 cells were lentivirally transduced with green fluorescence protein (GFP)‐epitope‐tagged wild‐type NICD1 or NICD1 (T2511A). Shown are immunoblots of the GFP‐epitope‐tagged wild‐type or mutated protein. (D) SNU‐449 cells transduced with GFP‐epitope‐tagged wild‐type NICD1 or NICD1 (T2511A) as in (C) were immunoprecipitated with anti‐GFP or anti‐IgG (control), and the lysate was immunoblotted with anti‐FBXW7 or anti‐GFP. (E) SNU‐449 cells were lentivirally transduced with GFP‐epitope‐tagged NICD1 or NICD1 (T2511A) without or with the shRNA‐mediated knockdown of *ASPM*‐v1 expression in the presence of MG132 (10 μm for 12 h). The poly‐ubiquitinated wild‐type or mutated NICD1 and the total immunoprecipitated GFP‐tagged proteins were detected by immunoblotting. β‐tubulin was included as a loading control. (F) The immunoblots of GFP or ASPM‐i1 in the total lysate of SNU‐449 cells transduced with GFP‐epitope‐tagged NICD1 or NICD1 (T2511A) without or with shRNA‐mediated knockdown of *ASPM*‐v1 expression. β‐tubulin was included as a loading control and the protein abundance levels of β‐tubulin were quantified by densitometric analysis of the bands in (A) to (F). Two lentivirus shRNA constructs (constructs #3 and #4) were used in the knockdown of *ASPM*‐v1 expression. The results represent two independent experiments in (A) to (F).

To gain further mechanistic insights into how ASPM‐i1 regulates the protein stability of NICD1, we performed co‐IP studies to demonstrate the strong association of ASPM‐i1 with NICD1 in HCC cells (Fig. 4A). Since ASPM‐i1 has been shown to compete with specific E3‐ubiquitin ligases for the binding to cyclin E, DVL, or BRCA1 and thereby interfere with their proteasome‐dependent degradation [18, 19, 21, 34, 35], we posited that ASPM‐i1 might regulate the stability of NICD1 by inhibiting its binding to specific E3‐ubiquitin ligases. It has been shown that the protein stability of NICD1 is mainly regulated by the E3‐ubiquitin ligase FBXW7 (also known as SEL10) [39, 40]. Concordantly, the knockdown of *ASPM*‐v1 expression triggered the recruitment of FBXW7 to the immunoprecipitated NICD1 in SNU‐449 cells (Fig. 4B). To corroborate the role of FBXW7‐mediated ubiquitination in ASPM‐i1‐regulated NICD1 stability, we mutated threonine 2511 of human NICD1, which mediates its interaction with FBXW7 [41], to alanine, and stably overexpressed wild‐type NICD1 or NICD1 (T2511A) in SNU‐449 cells using lentivirus‐mediated gene transduction (Fig. 4C). We confirmed that, unlike the wild‐type protein, NICD1 (T2511A) failed to associate with FBXW7 (Fig. 4D). Importantly, the knockdown of *ASPM*‐v1 expression prominently enhanced the poly‐ubiquitination of the overexpressed wild‐type NICD1. In sharp contrast, downregulating *ASPM*‐v1 expression failed to enhance the poly‐ubiquitination of the mutated NICD1 (T2511A) (Fig. 4E). Consistently, while the knockdown of *ASPM*‐v1 expression substantially reduced the protein abundance level of wild‐type NICD1, the protein abundance level of NICD1 (T2511A) remained stable in *ASPM*‐v1‐deficient cells (Fig. 4F). Collectively, these molecular studies lend support to a model in which ASPM‐i1 competes with FBXW7 for the binding to NICD1 and thereby prevents its proteasomal degradation.

### 3.4 NICD1 functionally rescues ASPM‐i1 deficiency in HCC cells

**Fig. 5:**
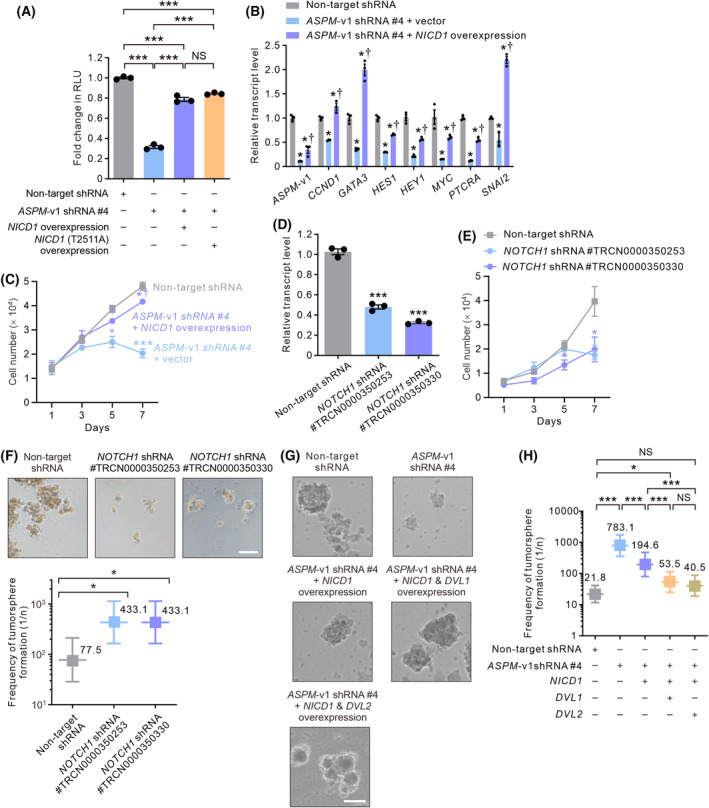
NOTCH1 intracellular domain (NICD1) and dishevelled (DVL) overexpression restored the tumorigenic potential of ASPM‐i1‐deficient HCC cells. (A) Relative Notch‐specific luciferase activity in SNU‐449 cells without (non‐target shRNA) or with shRNA‐mediated knockdown of *ASPM*‐v1 expression along with the lentivirus‐mediated overexpression of NICD1 or NICD1 (T2511A) (*n* = 3 independent experiments). Data are shown as mean ± SEM. ***P < 0.001, NS, not significant, two‐tailed unpaired *t* test. (B) The relative transcript levels of representative Notch target genes in SNU‐449 cells transduced with non‐target shRNA or *ASPM*‐v1 shRNA (construct #4) without (vector) or with the concurrent overexpression of NICD1 following stimulation with JAG1‐Fc (5 μg·mL^−1^ for 24 h) as measured by qRT‐PCR (*n* = 3 independent experiments). Data are shown as mean ± SEM and statistically analyzed by two‐tailed unpaired *t* test. *P < 0.05, compared with non‐target shRNA; ^†^ *P* < 0.05, compared with *ASPM*‐v1 shRNA #4 plus vector. (C) Line graphs showing the number of SNU‐449 cells without (non‐target shRNA) or with shRNA‐mediated knockdown of *ASPM*‐v1 expression without (vector) or with the lentivirus‐mediated NICD1 overexpression (*n* = 3 independent experiments). Data are shown as mean ± SEM, and statistically analyzed by two‐tailed unpaired *t* test. *P < 0.05, ***P < 0.001 compared with non‐target shRNA. (D) The relative transcript level of *NOTCH1* in SNU‐449 cells without (non‐target shRNA) or with shRNA‐mediated knockdown of *NOTCH1* expression as measured by qRT‐PCR (*n* = 3 independent experiments). Two lentivirus shRNA constructs (TRCN0000350253 and TRCN0000350330) were used for the genetic knockdown. (E) Line graphs showing the number of SNU‐449 cells lentivirally transduced with a non‐target shRNA or the *NOTCH1*‐targeted shRNA as in (D) (*n* = 4 independent experiments). Data are shown as mean ± SEM. *P < 0.05, ***P < 0.001 compared with non‐target shRNA in (D) and (E), two‐tailed unpaired *t* test. (F) Representative phase‐contrast images of the tumorspheres formed by SNU‐449 cells without (non‐target shRNA) or with shRNA‐mediated knockdown of *NOTCH1* expression as in (D). Scale bar denotes 100 μm (top). Limiting dilution assay demonstrating the tumorsphere‐forming efficacy (bottom) (*n* = 5 independent experiments). (G) Representative phase‐contrast images of tumorspheres formed by SNU‐449 cells without (non‐target shRNA) or with shRNA‐mediated knockdown of *ASPM*‐v1 expression without or with the lentivirus‐mediated overexpression of NICD1, NICD1, and DVL1, or NICD1 and DVL2. Scale bar denotes 100 μm. (H) Limiting dilution assay demonstrating the tumorsphere‐forming efficacy of the cells in (G). Bars represent maximum likelihood estimates with a 95% confidence interval (*n* = 16, control group; *n* = 8, experimental groups). *P < 0.05, ***P < 0.001, NS, not significant, the likelihood ratio test and Chi‐square test in (F) and (H).

Having demonstrated that ASPM‐i1 critically contributes to the Notch pathway activity by regulating the stability of NICD1 in HCC cells, we next investigated if NICD1 plays a crucial role in ASPM‐i1‐mediated HCC tumorigenesis. Indeed, the forced overexpression of NICD1 or NICD1 (T2511A) both markedly enhanced the Notch reporter activity in *ASPM*‐v1‐deficient SNU‐449 cells (Fig. 5A). Consistently, the overexpression of NICD1 significantly restored the expressions of Notch target genes (Fig. 5B). We then conducted a series of functional studies to affirm the oncogenic role of ASPM‐i1‐regualted NICD1 expression in HCC cells. First, we showed that the knockdown of *ASPM*‐v1 expression substantially reduced the proliferation of SNU‐449 cells (Fig. S3A) without inducing apoptosis (Fig. S3B,C). Importantly, NICD1 overexpression significantly restored the proliferative potential of *ASPM*‐v1‐deficient SNU‐449 cells (Fig. 5C). Echoing the pathogenetic role of NOTCH1 in HCC as implicated by clinical correlative studies [11, 13, 14], the genetic knockdown of *NOTCH1* expression crippled the proliferative potential of SNU‐449 cells and their ability to form tumorspheres (Fig. 5D–F). Conversely, the overexpression of NICD1 partially but significantly enhanced the tumorsphere‐forming capability of *ASPM*‐v1‐deficient cells (Fig. 5G,H). Furthermore, the concurrent overexpression of NICD1 and the upstream Wnt pathway regulator DVL1 or DVL2, which was reported to mediate the ASPM‐augmented Wnt pathway activity in HCC cells [20, 42], could further enhance the tumorigenic potential of *ASPM*‐v1‐deficient HCC cells (Fig. 5G,H). Together, these findings suggest that the Notch and Wnt signaling pathways cooperatively contribute to ASPM‐i1‐regulated tumorigenic potential of HCC cells.

### 3.5 NICD1 strongly co‐expresses with ASPM‐i1 in HCC cells

**Fig. 6:**
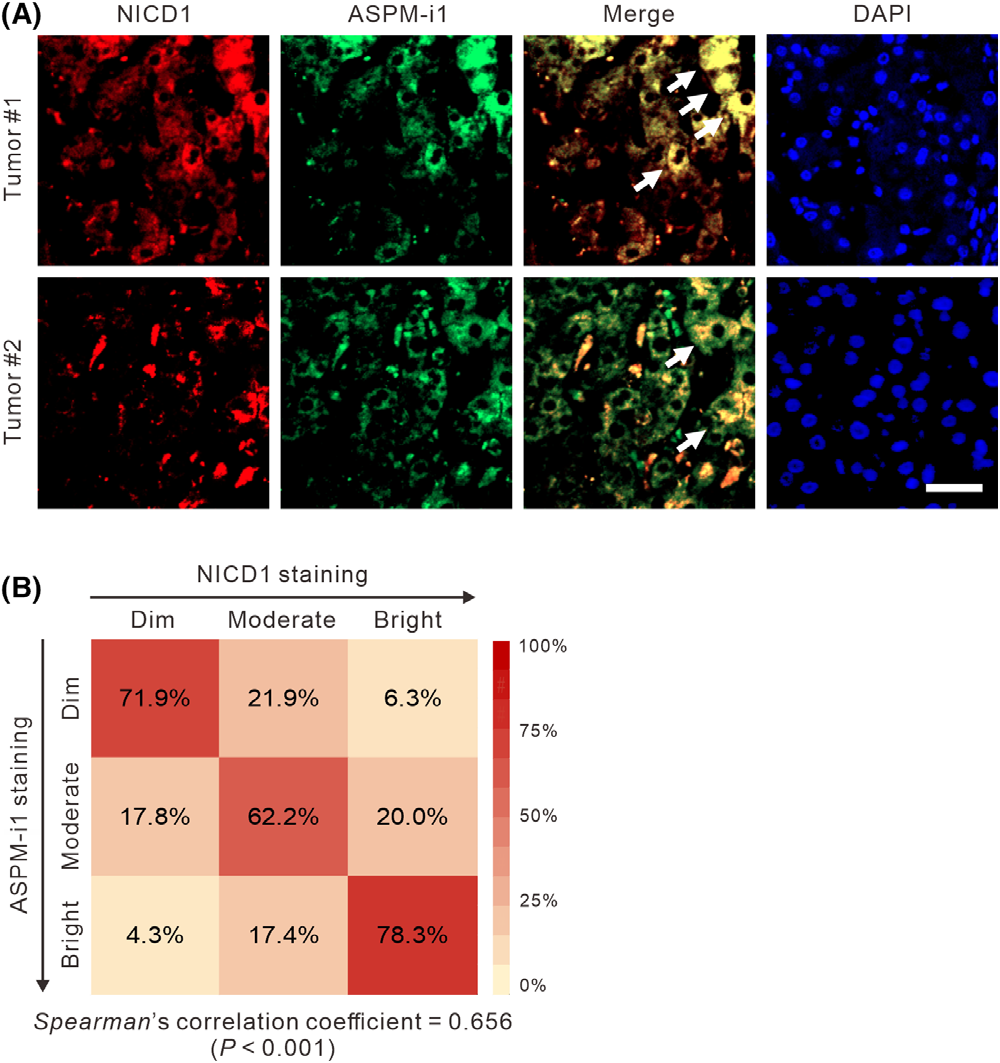
NOTCH1 intracellular domain (NICD1) co‐expresses with ASPM‐i1 in HCC cells. (A) Representative immunofluorescence images showing the marked co‐expression (yellow; arrows) of NICD1 (red) and ASPM‐i1 (green) in a subset of cancer cells in human HCC tissues. Nuclei were counterstained with DAPI (blue). Scale bar denotes 20 μm. (B) Heatmaps illustrating the correlation of the staining intensity of NICD1 and ASPM‐i1 at the single‐cell level in the human HCC tissues as in (A). *n* = 10 tumors; 300 cells per tumor counted.

**Fig. 7:**
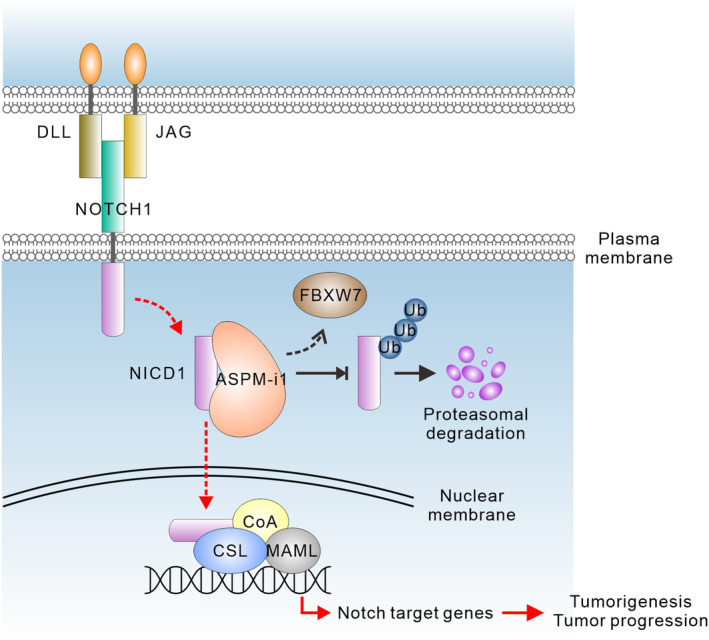
Molecular model depicting the NICD1/FBXW7/ASPM‐i1 regulatory module in HCC cells. The molecular antagonism between the E3‐ubiquitin ligase FBXW7 and ASPM‐i1 attenuates the poly‐ubiquitination of NICD1, thereby inhibiting its proteasomal degradation. The stabilized NICD1 then translocates into cell nuclei and renders HCC cells responsive to stimulation by Notch ligands, such as JAG1, resulting in the transcription of Notch pathway target genes and HCC tumorigenesis and progression. ASPM‐i1, abnormal spindle‐like microcephaly‐associated protein isoform 1; CoA, coactivator; CSL, CBF1, Suppressor of Hairless, Lag‐1; DLL, Delta‐like ligand; FBXW7, F‐box/WD repeat‐containing protein 7; HCC, hepatocellular carcinoma; JAG, Jagged; MAML, Mastermind‐like; NICD1, NOTCH1 intracellular domain; Ub, ubiquitin.

The crucial role of ASPM‐i1 in augmenting the protein stability of NICD1 in HCC cells raised the possibility that their respective expression may correlate with each other in HCC cells in human tumor tissues. To address this possibility, we undertook a co‐immunofluorescence analysis of NICD1, using a cleaved‐NOTCH1‐specific antibody, and ASPM‐i1 staining in HCC tissues at the single‐cell level. Echoing the expressional heterogeneity of ASPM‐i1 in HCC tissues (Fig. 1B), we uncovered a subset of cancer cells displaying moderate‐to‐bright ASPM‐i1 staining (Fig. 6A). Importantly, the staining intensity of ASPM‐i1 significantly correlates with that of NICD1 (*Spearman* correlation coefficient = 0.656; *P* < 0.001; Fig. 6B). The collective evidence supports the important role of the novel NICD1/FBXW7/ASPM‐i1 regulatory module in Notch signaling and HCC tumorigenesis (Fig. 7).

## 4 Discussion

Development signaling pathways, such as the Notch and Wnt signaling, regulate stem/progenitor cell states in adult tissues and are frequently dysregulated in malignant tumors, potentially giving rise to their oncogenic properties [7, 43]. In HCC, Wnt signaling is activated through multiple routes, including mutations in the β‐catenin (*CTNNB1*) or the *AXIN1* gene and the dysregulated expressions of Wnt receptors, ligands, and/or antagonists [44, 45, 46, 47]. Notably, many human HCCs with Wnt activation do not have *CTNNB1* mutations [15, 45]. Likewise, while approximately 30% of human HCC harbors Notch pathway activation, mutations in *NOTCH1* occur at a low frequency, and there is little evidence for genetic alterations in Notch signaling [7, 12, 15]. The compelling questions raised by these observations are how developmental pathways are intrinsically activated in HCC cells, and whether their activities are co‐regulated. One possible explanation is that specific regulatory hubs exist in HCC cells to coordinate the activities of multiple developmental signaling pathways. In this regard, ASPM‐i1 may serve as a regulatory hub in HCC as its expression was found to be indispensable for Notch and Wnt pathway activities and critically contributes to the oncogenic potential of HCC cells according to our studies [20].

Mounting data accumulated over recent years suggested that ASPM plays an essential role in the oncogenesis of various human cancers [18, 19, 20, 21, 23]. In HCC, ASPM has been implied as a hub of tumorigenesis according to bioinformatics and clinical correlative analyses [48, 49]. A large‐scale transcript analysis revealed the overexpression of *ASPM* in HCC tissues and the significant association of its expression with early tumor recurrence and the survival of patients with HCC [25]. A recent study further identified the considerable cell‐to‐cell heterogeneity in ASPM expression within HCC tissues [20]. Notwithstanding these findings, the molecular mechanism behind ASPM and ASPM‐i1 overexpression in HCC remains unclear. In glioblastoma and gastric cancer, ASPM overexpression is not due to changes in the methylation level of its promoter or copy number gain but is mediated by the mutant EGFR (EGFRvIII) signaling [50] or the transcriptional factor FOXM1 [19, 51]. Whether or not mutant EGFR or FOXM1 also regulates the expression of ASPM‐i1 in HCC cells remains unclear, and additional screening strategies will be required to identify the upstream signaling pathways underlying ASPM overexpression.

The *ASPM* gene has several putative transcript variants, which encode protein isoforms that vary considerably in size and subcellular localization [17]. As such, simply measuring the total transcript or protein abundance level of *ASPM* may potentially lead to a biased interpretation of its expression pattern in tumors. Our study revealed the specific upregulation in the expression of ASPM‐i1 in HCC cells and supported its expression level as a prognosticator in HCC. We further demonstrated the essential role of ASPM‐i1 in regulating the Notch signaling pathway activity in HCC cells. Aside from Notch signaling, ASPM also regulates the Wnt‐β‐catenin signaling pathway in HCC cells [20, 42]. Thus, it can be envisaged that ASPM‐i1^high^ HCC cells may preferentially respond to therapeutics targeting these developmental pathways, yielding a novel therapeutic opportunity for HCC.

The oncogenic function of Notch signaling has been attributed to its role in various aspects of tumor biology, including cell cycle progression, apoptosis, angiogenesis, epithelial–mesenchymal transition, and cancer stemness [7]. Once the Notch signaling pathway becomes activated, NICD is translocated into the nucleus, which then transcriptionally upregulates the expression of a core set of genes, including *HES*, *HEY*, *MYC*, *CCND1*, *GATA3*, and *PTCRA* [30, 52, 53, 54, 55]. Mounting studies have shown that Notch target genes are involved in the regulation of the differentiation and development of liver tissues as well as HCC tumorigenesis and tumor progression. For instance, the NOTCH‐HES1 axis was found to be critical in the development of HCC via hepatitis B virus X protein [56]. HES1 was shown to regulate cell proliferation and migration of HCC cells, as well as the activation of the EMT program [57, 58]. HEY1 expression is upregulated in HCC [59], wherein it maintains tumor‐initiating cells [60]. HEY1 also promotes HCC cell proliferation, migration, and tumor growth [61, 62]. Furthermore, the Notch1‐MYC‐VCAM1 signaling axis was recently reported to initiate hepatocarcinogenesis, macrophage‐dependent trans‐endothelial migration, and metastasis [13].

Of particular relevance to our study is the increasingly appreciated role of Notch signaling in the maintenance of stem‐like cancer cells in cancers. For instance, it has been reported that CD44^+^ gastric cancer cells express a high level of the Notch target gene *HES1*, and the Notch inhibitor γ‐secretase inhibitor could effectively inhibit their malignant properties by concomitantly inhibiting the expressions of Notch and Wnt pathway genes [63]. Overexpression of NICD1 transforms rat liver progenitor cells into highly tumorigenic stem‐like cancer cells [13]. Similarly, γ‐secretase inhibitor could reduce the growth of glioma tumorspheres and deplete stem‐like cancer cells *in vivo* [64]. Echoing these findings, our study identifies ASPM‐i1 as the critical regulator of NICD1 in Notch pathway active HCC cells, raising the possibility that ASPM‐i1^high^NICD1^high^ cells may consist of the enriched population of stem‐like cells in HCC. We additionally demonstrated that Notch and Wnt signaling cooperatively contribute to ASPM‐i1‐mediated HCC tumorigenesis as the concurrent overexpression of NICD1 and DVL1 or DVL2 could rescue the tumorigenic potential of ASPM‐deficient cancer cells. It is noteworthy that multiple lines of studies have reported the reciprocal crosstalk between Wnt and Notch signaling in cancer cells mediated through diverse mechanisms involving protein–protein interactions and transcriptional regulations [38, 63, 65, 66], leaving open the question of whether NICD1, DVL, and/or β‐catenin also establish molecular interactions in ASPM‐i1^high^ HCC cells. If so, the epistatic relationship between Notch and Wnt pathways and their respective contribution to HCC tumorigenesis warrants further investigations.

Previously, our group has demonstrated that ASPM regulates the tumorigenic potential of a subset of HCC cells by inhibiting the protein degradation of DVL1, a cardinal upstream regulator of Wnt signaling [20]. We have recently identified the region where DVL proteins interact with ASPM, which is located within the region encoded by exon 18 (data not shown). Notably, the exon‐18‐encoded region is missing in ASPM‐i2 and the other putative ASPM isoforms [17, 18]. This provides a plausible explanation of why ASPM‐i1 specifically regulates DVL proteins in cancer cells and why malignant cells preferentially upregulate the expression of ASPM‐i1 in HCC cells and other types of cancer cells [18, 19]. Since ASPM‐i1, but not ASPM‐i2, specifically binds to NICD1 in HCC cells, it is likely that ASPM may also interact with NICD1 through the ASPM‐i1‐specific exon‐18‐encoded region. This likelihood can be addressed by mapping the domains on ASPM‐i1 that mediate its interaction with NICD1, which is currently underway.

## 5 Conclusions

The current study identified the interaction between NICD1, FBXW7, and ASPM‐i1 as a novel and crucial molecular mechanism underlying the heightened Notch signaling activity in HCC. Our findings add the Notch pathway to the growing list of molecular pathways that are regulated by the pleiotropic scaffold protein ASPM‐i1 and imply that it contributes to HCC tumorigenesis and progression by positively co‐regulating Notch and Wnt signaling. Our data, thus, illuminate an important regulatory module in HCC tumorigenesis, representing a targetable vulnerability that may unlock a new opportunity for improving the outcome of patients with HCC.

## Conflict of interest

KKT has a patent for the Patent Cooperation Treaty application pending to Taipei Medical University. The remaining authors declare no competing interests.

## Author contributions

T‐SC, L‐HC, C‐CH, T‐YL, P‐CL, W‐CH, and C‐PC conducted molecular and biochemical experiments. H‐YH conducted animal studies. T‐SC and C‐CH performed and analyzed immunostaining. T‐SC compiled clinical data and performed the statistical analysis. P‐MY conducted bioinformatics analyses. KKT acquired funding, supervised the research, and prepared the manuscript. C‐PC revised the manuscript. All authors have read and approved the final version of the manuscript.

### Peer review

The peer review history for this article is available at https://www.webofscience.com/api/gateway/wos/peer‐review/10.1002/1878‐0261.13589.

## Supporting information


**Fig. S1.** ASPM isoform 1 (ASPM‐i1) contributes to the protein expression level of NICD1 without affecting the transcript level of *NOTCH1* in HCC cells.
**Fig. S2.** The knockdown of *ASPM* variant 1 (*ASPM*‐v1) expression reduced the stability of NOTCH intracellular domain 1 (NICD1) in Notch signaling activated HCC cells.
**Fig. S3.** Downregulating *ASPM* variant 1 (*ASPM*‐v1) expression reduced the proliferative potential of HCC cells without inducing apoptosis.
**Table S1.** A list of antibodies used in the study.

## Data Availability

The data sets obtained and/or analyzed in the current study are available from the corresponding author upon reasonable request.
