# Supplementary material for: ASPM stabilizes the NOTCH intracellular domain 1 and promotes oncogenesis by blocking FBXW7 binding in hepatocellular carcinoma cells
Source: Mol Oncol. 2024 Jan 26;18(3):562–79. doi: 10.1002/1878-0261.13589 (PMC10920086; doi:10.1002/1878-0261.13589)
Supplement: Supplementary file 1 — Fig. S1. ASPM isoform 1 (ASPM‐i1) contributes to the protein expression level of NICD1 without affecting the transcript level of NOTCH1 in HCC cells. Fig. S2. The knockdown of ASPM variant 1 (ASPM‐v1) expression reduced the stability of NOTCH intracellular domain 1 (NICD1) in Notch signaling activated HCC cells. Fig. S3. Downregulating ASPM variant 1 (ASPM‐v1) expression reduced the proliferative potential of HCC cells without inducing apoptosis. Table S1. A list of antibodies used in the study. [file MOL2-18-562-s001.docx]

**ASPM stabilizes the NOTCH intracellular domain 1 and promotes oncogenesis by blocking FBXW7 binding in hepatocellular carcinoma cells**

Tze-Sian Chan, Li-Hsin Cheng, Chung-Chi Hsu, Pei-Ming Yang, Tai-Yan Liao, Hsiao-Yen Hsieh, Pei-Chun Lin, Wei-Chun HuangFu, Chih-Pin Chuu, Kelvin K. Tsai

**Figure S1**

**
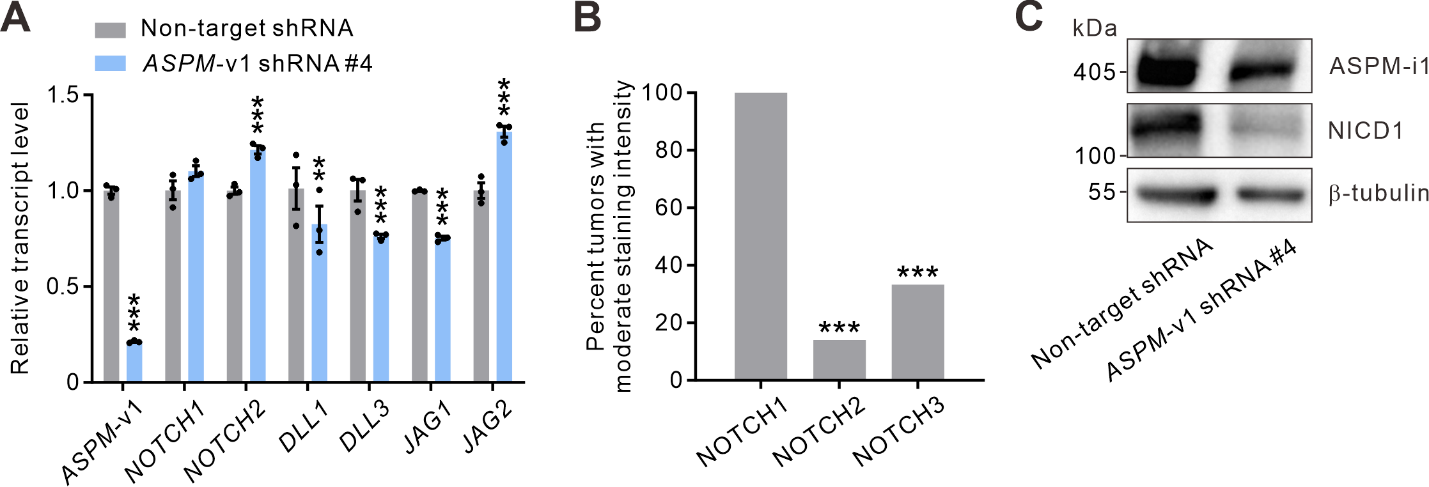
**

**Figure S1. ASPM isoform 1 (ASPM-i1) contributes to the protein expression level of NICD1 without affecting the transcript level of *NOTCH1* in HCC cells.** (A) The transcript level of the Notch pathway ligands and receptors in SNU-449 cells without (non-target) or with shRNA-mediated knockdown of *ASPM* variant 1 (*ASPM*-v1) expression. The transcript levels of *NOTCH3* and *DLL4* are not included due to their high cycle threshold values (> 30) (*n* = 3 independent experiments). Data are shown as mean ± SEM. ***P* < 0.01, ****P* < 0.001 compared with non-target shRNA, two-tailed unpaired *t* test. (B) The percentage of tumors exhibiting a moderate or a high staining intensity of NOTCH1, NOTCH2, or NOTCH3 in human HCC tissues according to the Human Protein Atlas data sets (https://www.proteinatlas.org/). ****P* < 0.001 compared with NOTCH1, Fisher’s exact test. (C) Immunoblots of ASPM-i1 and NOTCH1 intracellular domain (NICD1) in SNU-449 cells without (non-target) or with shRNA-mediated knockdown of *ASPM*-v1 expression. β-tubulin was included as the loading control (*n* = 2 independent experiments). The lentivirus *ASPM*-v1 shRNA construct #4 was used to knock down *ASPM*-v1 expression in (A) and (C).

**Figure S2**

**
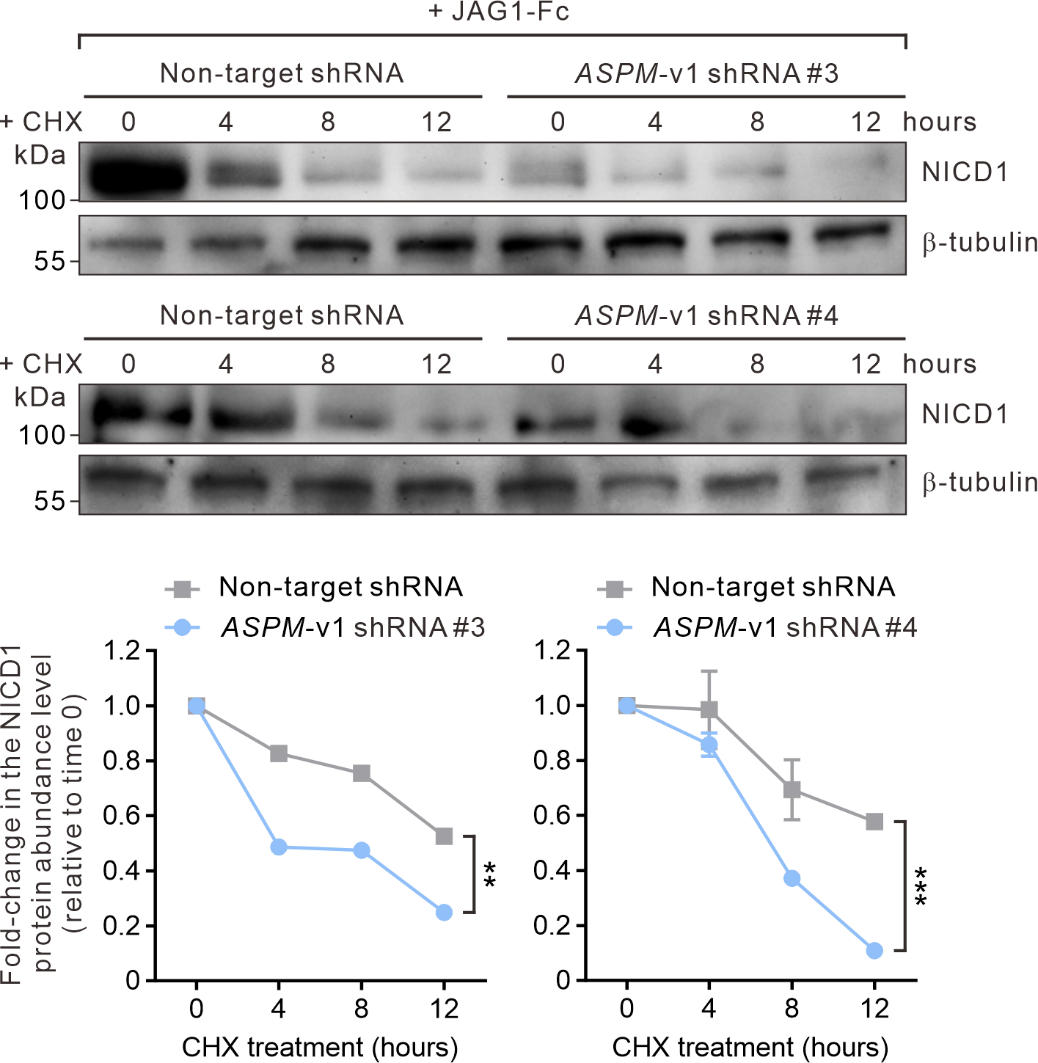
**

**Figure S2. The knockdown of *ASPM* variant 1 (*ASPM*-v1) expression reduced the stability of NOTCH intracellular domain 1 (NICD1) in Notch signaling activated HCC cells.** Shown are the immunoblots of NICD1 in SNU-449 cells lentivirally transduced with *ASPM*-v1 shRNA (construct #3 or #4) or a non-target shRNA and treated with the Notch ligand JAG1-Fc (5 µg/ml for 24 hours) and then cycloheximide (CHX) for the indicated length of time (top). Line graphs demonstrating the time-dependent change in the NICD1 protein levels (bottom). β-tubulin was included as a loading control (*n* = 2 independent experiments). Data are shown as mean ± SEM. ***P* < 0.01, ****P* < 0.001, ordinary two-way ANOVA**.**

**Figure S3**

**
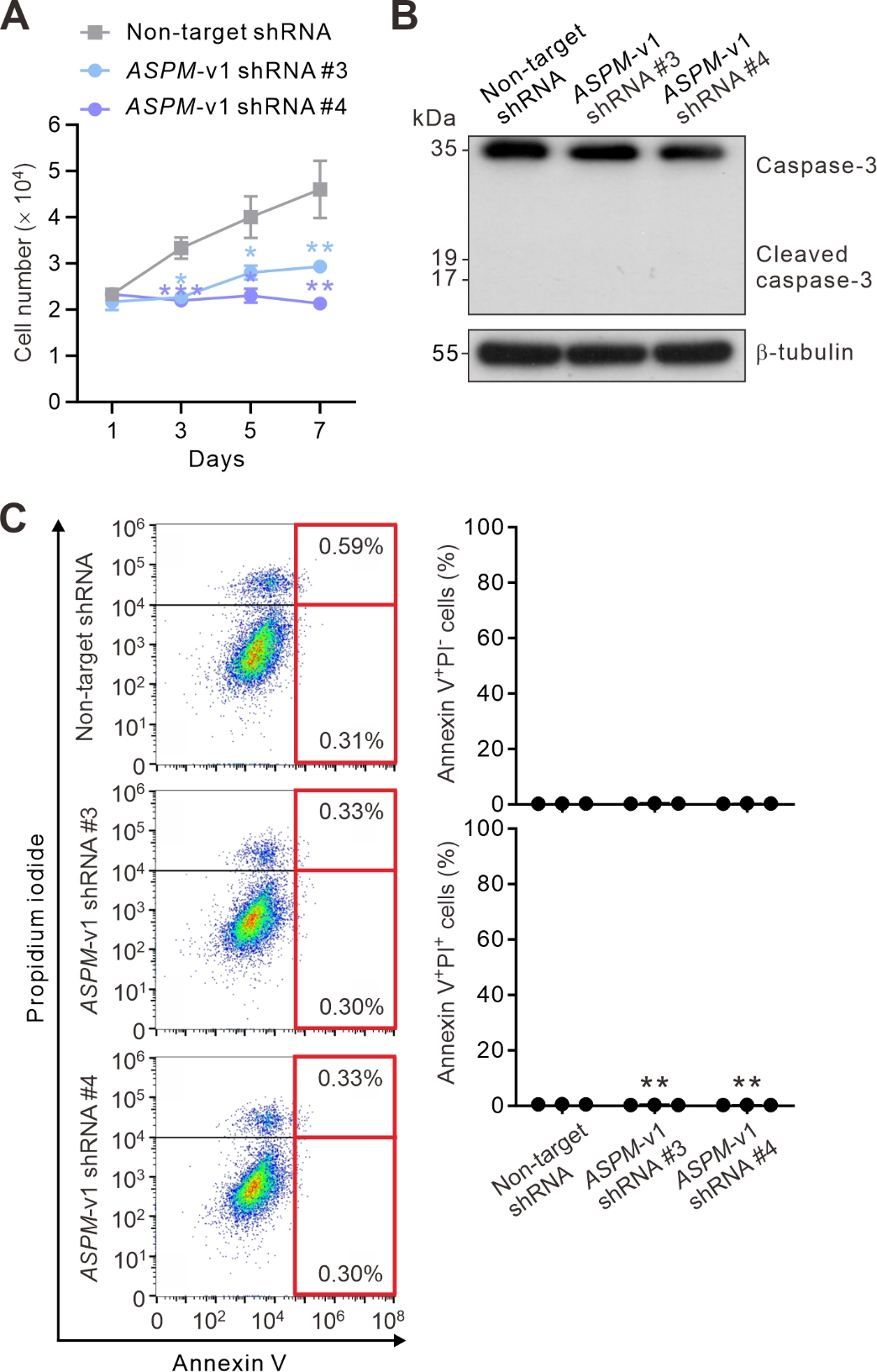
**

**Figure S3.** **Downregulating** ***ASPM* variant 1 (*ASPM*-v1) expression reduced the proliferative potential of HCC cells without inducing apoptosis.** (A) Line graphs showing the number of SNU-449 HCC cells lentivirally transduced with a non-target shRNA or *ASPM*-v1 shRNA. Two lentivirus shRNA constructs (constructs #3 and #4) were used for the genetic knockdown of *ASPM*-v1 (*n* = 3 independent experiments). (B) Immunoblots of total and cleaved caspase 3 in control (non-target shRNA) or *ASPM*-v1 knocked down SNU-449 cells as described in (A). β-tubulin was included as a loading control (*n* = 2 independent experiments). (C) Representative flow cytometry plots showing the percentage of Annexin V-positive and propidium iodide (PI)-negative cells (Annexin V^+^PI^-^ cells; representing early apoptotic cells) or Annexin V^+^PI^+^ cells (representing late apoptotic cells) in control or *ASPM*-v1 knocked down SNU-449 cells as described in (A) (*n* = 12 independent experiments). Data are shown as mean ± SEM. **P* < 0.05, ***P* < 0.01, ****P* < 0.001 compared with non-target shRNA in (A) and (C).

**Supplementary Table S1. A list of antibodies used in the study**

| Reagent or resource | Source | Identifier |
| --- | --- | --- |
| Rabbit polyclonal anti-ASPM isoform 1 | This study^1^ |  |
| Rabbit polyclonal anti-ASPM-isoform 2 | This study^1^ |  |
| Rabbit polyclonal anti-β-tubulin | GeneTex | Cat #GTX101279  RRID: AB_1952434 |
| Rabbit polyclonal anti-β-catenin | GeneTex | Cat #GTX101435 RRID: AB_1950062 |
| Rabbit polyclonal anti-caspase 3 | GeneTex | Cat #GTX110543  RRID: AB_10722709 |
| Mouse monoclonal anti-FBXW7 | Thermo Fisher Scientific | Clone OTI4C11, Cat #MA5-26563  RRID: AB_ 2724297 |
| Mouse monoclonal anti-GFP | Santa Cruz | Clone B-2, Cat #sc-9996  RRID: AB_ 627695 |
| Rabbit monoclonal anti-cleaved Notch1 (Val1744) | Cell signaling | Clone D3B8, Cat #4147  RRID: AB_2153348 |
| Rabbit polyclonal anti-ubiquitin | GeneTex | Cat #GTX128826  RRID:AB_2885822 |

^1^C. C. Hsu *et al.*, The differential distributions of ASPM isoforms and their roles in Wnt signaling, cell cycle progression, and pancreatic cancer prognosis. *J Pathol* **249**, 498-508 (2019).

**References:**

1 Liao WY, Hsu CC, Chan TS, Yen CJ, Chen WY, Pan HW *et al*. Dishevelled 1-Regulated Superpotent Cancer Stem Cells Mediate Wnt Heterogeneity and Tumor Progression in Hepatocellular Carcinoma. *Stem Cell Reports* 2020; 14: 462-477.
